# Ethics and the marketing authorization of pharmaceuticals: what happens to ethical issues discovered post-trial and pre-marketing authorization?

**DOI:** 10.1186/s12910-020-00543-w

**Published:** 2020-10-27

**Authors:** Rosemarie D. L. C. Bernabe, Ghislaine J. M. W. van Thiel, Nancy S. Breekveldt, Christine C. Gispen, Johannes J. M. van Delden

**Affiliations:** 1 Faculty of Health and Social Sciences, University of South-Eastern Norway, Kongsberg, Norway; 2 Centre for Medical Ethics, Institute of Health and Society, University of Oslo, Oslo, Norway; 3 Julius Center for Health Sciences and Primary Care, University Medical Center Utrecht, Utrecht, The Netherlands; 4 Dutch Medicines Evaluation Board, Utrecht, The Netherlands

## Abstract

**Background:**

In the EU, clinical assessors, rapporteurs and the Committee for Medicinal Products for Human Use are obliged to assess the ethical aspects of a clinical development program and include major ethical flaws in the marketing authorization deliberation processes. To this date, we know very little about the manner that these regulators put this obligation into action. In this paper, we intend to look into the manner and the extent that ethical issues discovered during inspection have reached the deliberation processes.

**Methods:**

To gather data, we used the Dutch Medicines Evaluation Board database and first searched for the inspections, and their accompanying site inspection reports and integrated inspection reports, related to central marketing authorization applications (henceforth, application/s) of drugs submitted to the European Medicines Agency (EMA) from 2011 to 2015. We then extracted inspection findings that were purely of ethical nature, i.e., those that did not affect the benefit/risk balance of the study (issues related to informed consent, research ethics committees, and respect for persons). Only findings graded at least major by the inspectorate were included. Lastly, to identify how many of the ethically relevant findings (ERFs) reach the application deliberation processes, we extracted the relevant joint response assessment reports and reviewed the sections that discussed inspection findings.

**Results:**

From 2011 to 2015, there were 390 processed applications, of which 65 had inspection reports and integrated inspection reports accessible via the database of the Dutch Medicines Evaluation Board. Of the 65, we found ERFs in 37 (56.9%). The majority of the ERFs were graded as major and half of the time it was informed-consent related. A third of these findings were related to research ethics committee processes and requirements. Of the 37 inspections with ERFs, 30 were endorsed in the integrated inspection reports as generally GCP compliant. Day 150 joint response assessment reports and Day 180 list of outstanding issues were reviewed for all 37 inspections, and none of the ERFs were carried over in any of the assessment reports or list of outstanding issues.

**Conclusion:**

None of the ethically relevant findings, all of which were graded as major or critical in integrated inspection reports, were explicitly carried over to the joint assessment reports. This calls for more transparency in EMA application deliberations on how ERFs are considered, if at all, in the decision-making processes.

## Background

Several documents from the European Medicines Agency (EMA) speak of the place of ethics in the regulatory processes involved in a marketing authorization application (henceforth, application) [1–4]. One of these is the document, *Points to consider on Good Clinical Practice (GCP) inspection findings and the benefit-risk balance* where the mandate of regulators in terms of the place of these ethical issues in the evaluation process is explained as follows:

GCP inspection findings – *even if not directly influencing the benefit-risk balance*—will still be important if they raise serious questions about the rights, safety and well-being of trial subjects and hence the overall ethical conduct of the study. It is an obligation of clinical assessors, rapporteurs and the CHMP also to assess the ethics of a clinical development programme, and major ethical flaws should have an impact on the final conclusions about approvability of an application. Consequently, ethical misconduct could result in rejection of the application [4]. (*italics mine*).

In a previous publication, we identified the types of ethical issues that pharmaceutical regulators encounter post-marketing through inspection reports [5]. In this publication, we discovered that based on 2008–2012 inspection reports comprising of 112 medicinal products and 288 clinical trial sites, inspectors frequently and regularly encounter ethically relevant findings (ERFs). Specifically, "At least major ERFs were present in almost all medicinal products with ERFs. The categories with the highest number of ERFs were protocol issues, patient safety, and professionalism issues." Also, "on average, there were 7.54 major and 2.95 critical ERFs per medicinal product application, although ERFs can increase to 30 major and 12 critical" [5]. For more information on what inspectors consider as major and critical ERFs, the reader is directed to consult our article entitled, “Ethics in clinical trial regulation: ethically relevant issues from EMA inspection reports” [5]. Though it is fair to assume that at least some of the ERFs that “directly influence the benefit-risk balance” of an investigational medicinal product submitted for marketing authorization application would be carried over to the succeeding regulatory deliberation processes, we cannot make the same assumption about GCP inspection findings that do “not directly influence the benefit-risk balance.” The latter remains unknown and, as such, we know very little about the manner that “clinical assessors, rapporteurs and the Committee for Medicinal Products for Human Use (CHMP)” fulfill this obligation of “assessing the ethics of a clinical development programme.” To respond to this need, it is the goal of this article to look into the manner and extent that ethical issues that do not affect benefit-risk balanced and were discovered during inspection have reached the deliberation processes, i.e., how “major ethical flaws” have impacted “the final conclusions about (the) approvability of an application.”

## Methods

**Fig. 1:**
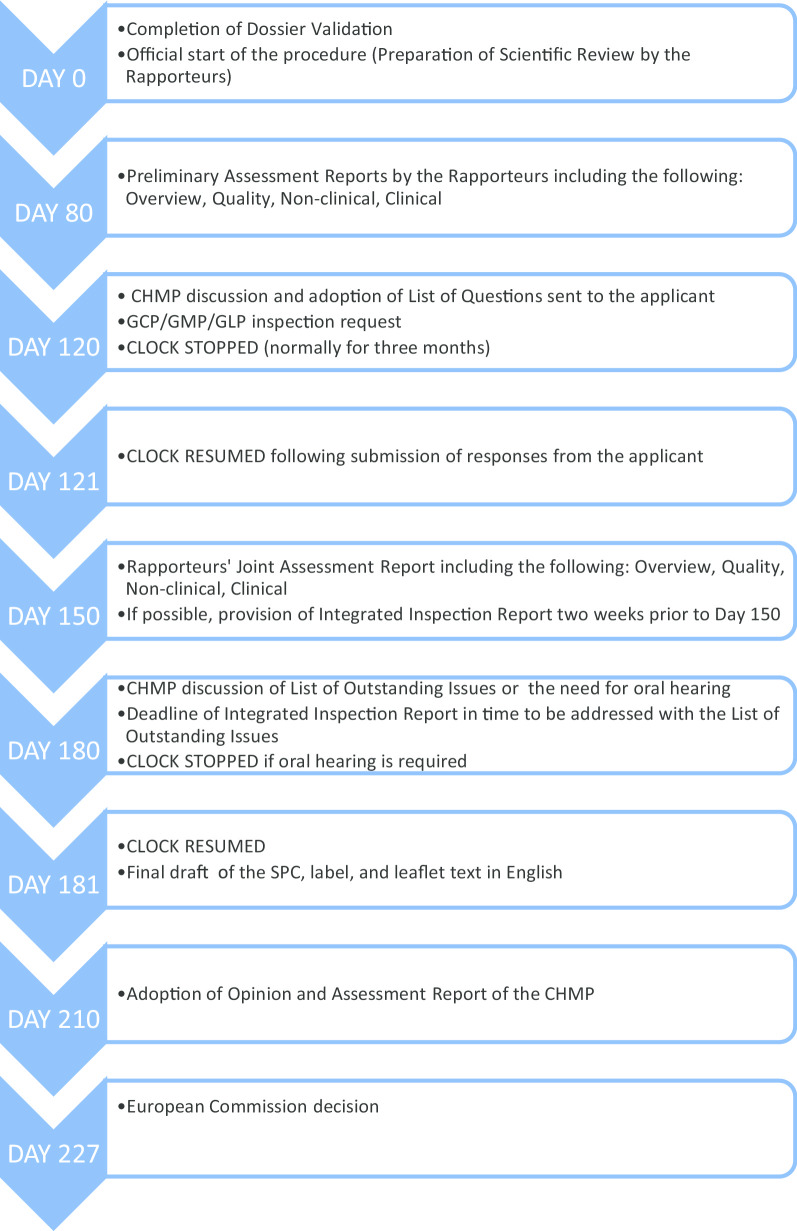
European centralized procedure for authorizing medicinal products [6–8]

Before we elaborate on our methodology, it is imperative that we quickly go through the European centralized procedure for authorizing medicinal products, which we have outlined in Fig. 1.

**Fig. 2:**
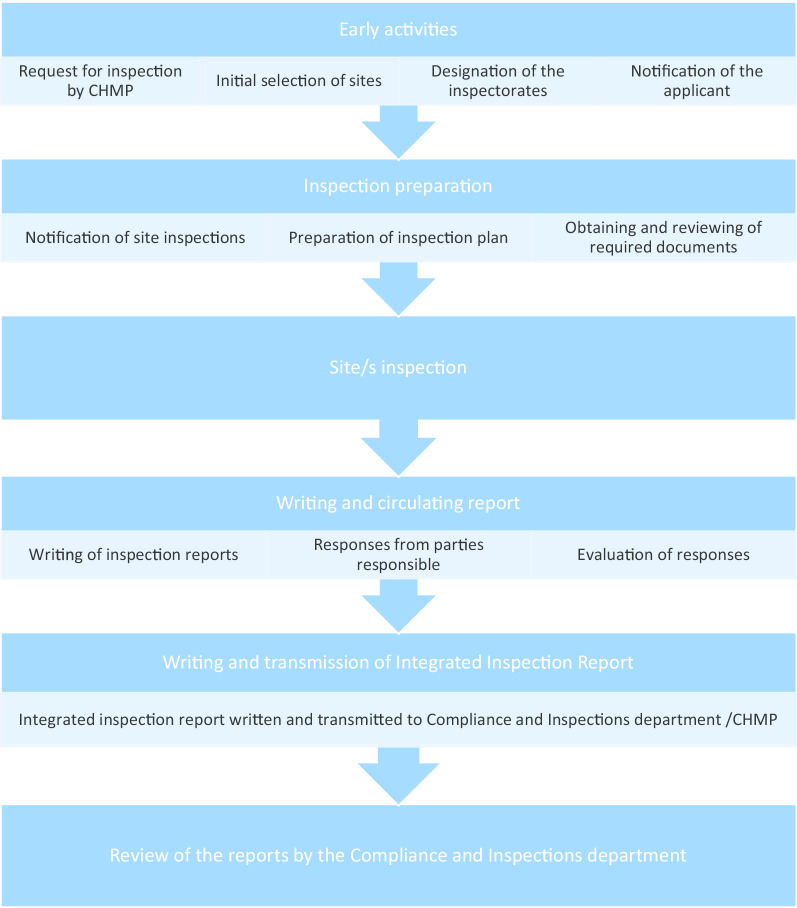
Process of inspection activities related to CHMP request [2, 3]

As can be seen from Fig. 1, the request for GCP inspections and the eventual circulation of the integrated inspection report to the CHMP happens between Day 120 and Day 180. All inspection reports and integrated inspection reports are submitted to the CHMP for the latter’s consideration. Figure 2 provides the details leading to the circulation of the integrated inspection report.

Given the centralized procedure outlined above, to understand the extent to which ethical issues have reached the application deliberation processes, we searched for *inspection reports, integrated inspection reports, Day 150 joint assessment reports, and Day 180 List of Outstanding Issues*.

To gather data, we used the Dutch Medicines Evaluation Board database and first searched for inspections, and their accompanying site inspection reports and integrated inspection reports, related to central application of drugs submitted to the EMA from 2011 to 2015. For the list of drugs processed for central marketing authorization, we used the European public assessments report database [9].

Inspection findings include both scientific and ethical issues. To determine which issues to extract, we used the following system. In another publication, we extracted the ethical issues from GCP inspection reports and came up with the following classifications of ethical issues: informed consent, monitoring and oversight, patient safety, professionalism and or qualification issues, protocol compliance or protocol issues, research ethics committees, and respect for persons [5]. It can be observed that the issues in some of the classifications can both be scientific and ethical. An ethical issue can also be a scientific issue when it could affect the benefit-risk balance of a scientific evaluation of an application [4]. The following classifications have this dual characteristic: monitoring and oversight, patient safety, professionalism and or qualification issues, protocol compliance or protocol issues. Since we wish to investigate the impact of an ethical issue that is not a scientific issue, we shall look at the issues under the following classifications only: informed consent, research ethics committees, and respect for persons. The former three classifications coincide with the list of ethical issues that may trigger a “for cause” inspection as stated in the document, *Points to consider for assessors, inspectors and EMA inspection coordinators* [1]. We used another of our publications [10] to define the scope of informed consent (*IC*), research ethics committees (*REC*), and respect for persons.

Even within the latter three categories, since we are testing how far purely ethical issues identified in inspections reach the evaluation processes, we excluded inspection findings that may influence the benefit-risk balance evaluation. For example, one of the issues identified as likely to influence benefit-risk evaluation is “inadequate reporting of adverse events and other safety endpoints.” If we look at the definition of respect for persons, patient safety is an aspect of its definition and inadequate reporting of (severe) adverse events a concrete example. Because this finding is likely to affect benefit-risk evaluation, i.e., it is clearly both a scientific and an ethical issue, it was excluded from our analysis.

The GCP deviation findings from inspection reports that were graded by the inspectors as either major or critical and that may be categorized under IC, REC, and/or respect for persons were extracted (henceforth ethically relevant findings, *ERFs*). We used the integrated inspection reports to validate if the inspection findings still hold after the evaluation of the responses of the responsible parties on the initial inspection reports (see Fig. 2) and if the gravity rating remains the same. In case of a discrepancy, we followed the integrated inspection reports. The conclusion from the integrated inspection reports were extracted.

Next, to identify how many ERFs reach the evaluation of the application, the relevant joint response assessment reports (specifically the documents “overview” and “clinical”) and the list of outstanding issues (see Fig. 1) were extracted. We reviewed the sections where these assessment reports discussed the inspection findings and identified if and how these ERFs were considered in the evaluation processes and how the issues ultimately affected the decision on the application.

To avoid privacy and confidentiality issues, the results are on an aggregated format.

## Results

From 2011 to 2015, 390 applications were processed, of which 65 had inspection reports and integrated inspection reports accessible via the database of the Dutch Medicines Evaluation Board. Of the 65, we found ERFs in 37 (56.9%). These findings are summarized in Table 1.

**Table 1:**
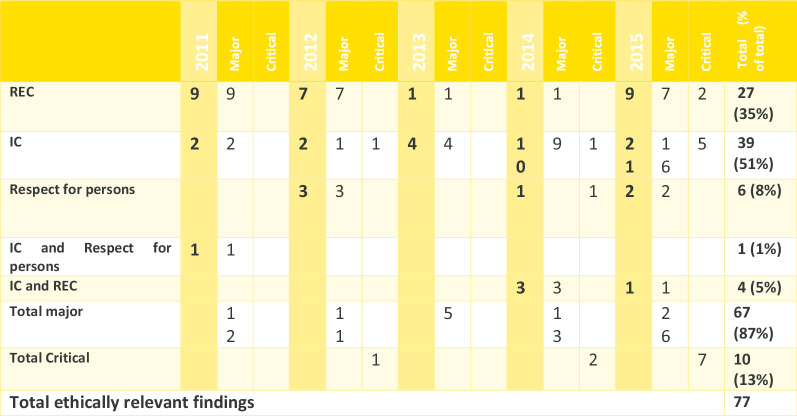
Grading and quantity of ethically relevant findings

As can be seen from Table 1, the majority of the ERFs were graded as major and half of the time it was IC-related. A third of these findings were related to research ethics committee processes and requirements.

Of the 37 inspections with ERFs, 30 were endorsed in the integrated inspection reports as generally GCP compliant. Table 2 presents the mean, mode, minimum, and maximum ERF values in all inspections, endorsed inspections (the 30 inspections), and not-fully-endorsed inspections (the remaining 7 inspections).

**Table 2:** Mean, mode, minimum, and maximum ERF values grouped according to all inspections, endorsed inspections, and non-endorsed inspections

	Total ERFs	Major ERFs	Critical ERFs
All Inspections (37)	n = 77	n = 67	n = 10
Mean	2.1	2.0	1.4
Mode	1	1	1
Minimum	4	4	1
Maximum	6	6	4

Endorsed inspections (30)	n = 56	n = 51	n = 5
Mean	3.6	1.9	1.0
Mode	1	1	1
Minimum	1	1	1
Maximum	4	4	1

Not-fully-endorsed Inspections (7)	n = 21	n = 6	n = 5
Mean	5.3	2.3	2.5
Mode	2	1	1
Minimum	1	1	1
Maximum	6	6	4

From Table 2, we see that there is a difference in terms of the average number of ERFs and the maximum number of ERFs per inspection between the endorsed and the non-endorsed inspections. Non-endorsed inspections have higher values on both counts than endorsed inspections in terms of total number of ERFs, major ERFs, and critical ERFs. This means that the non-endorsed inspections have more and graver ERFs than the endorsed inspections.

In all the 30 endorsed inspections, the gravity ratings were retained and the corrective and preventive action (CAPA) proposals of the sponsors and investigators to address the ERFs were accepted by the inspectors. Note that CAPAs would in most instances be preventive, i.e., changes can be made only for future trials. Seven of the inspections were not fully endorsed as GCP compliant, partly due to ERFs.

Of the seven not-fully-endorsed inspection cases, three were declared non-GCP compliant with the consequence that (part of) the data were not endorsed for use for the assessment of an application. One was declared non-GCP compliant but data were still endorsed for use during assessment. In three cases, data were endorsed for use for assessment, but the inspectors expressed lingering concerns about ERFs and required a better approach from the sponsor in the future.

Day 150 joint response assessment reports and Day 180 list of outstanding issues were reviewed for all 37 inspections, and none of the ERFs were carried over in any of the assessment reports or list of outstanding issues. Table 3 summarizes these results.

**Table 3:** Highlights

	*N*
Inspected marketing authorization applications with ethically relevant findings	37
Inspections with the conclusion that the inspected sites were generally GCP compliant	30
Ethically relevant findings discussed in Day 150 joint response assessment reports or Day 180 list of outstanding issues	0

## Discussion

In our study, we wanted to see how many of the ethical issues that were not likely to affect the scientific validity of the study and that were discovered during inspection have reached the evaluation processes for centralized applications of drugs. We did this by investigating how many of the ERFs from integrated inspection reports were reflected in Day 150 and Day 180 joint assessment reports. Our results are straightforward: of the 77 ERFs found in 56.9% of all inspections from 2011–2015, none of the ERFs were factored in, i.e., none of them were mentioned at all as factors to consider in either Day 150 joint response assessment reports or Day 180 list of outstanding issues. This means that though these ERFs may have been discussed internally, none of these were explicitly carried over to the joint assessment reports. Whether or not the inspections were endorsed was not a factor in the uptake of ERFs in Day 150 and Day 180 assessments. This is disturbing especially for the seven inspections where the inspectors did not guarantee general GCP compliance of the trial sites, three of which lingering concerns about ERFs were expressed by the inspectors. Overall, and based on inspection and assessment reports, this means that the mandate obliging clinical assessors, rapporteurs and the CHMP to also “assess the ethics of a clinical development programme, and major ethical flaws should have an impact on the final conclusions about approvability of an application” [1] have yet to be actualized or at least seen as factors explicitly considered during the assessment of an application. With that said, some considerations are worth mentioning.

First, it is unclear what standards inspectors use to declare that the inspected sites were generally GCP compliant in spite of major/critical ERFs. *Major/critical* issues are defined as “conditions, practices or processes that *might adversely/adversely* affect the rights, safety or well-being of the subjects and/or the quality and integrity of data” [11]. If major/critical ERFs at the very least have the possibility of affecting the rights, safety, or well-being of the subjects, how were these weighed and factored in the conclusion that the sites were generally GCP compliant? At the time of writing, we know of no EMA document that speaks about this process. Thus, there is a need for a transparent structure on grading standards as well as guidelines on the place of minor/major/critical findings in application decision-making.

Second, though the grading of critical/major/minor is used by the inspectors, it is not clear in EMA documents if the assessors should use the same grading system. Whether inspectors and assessors should and in fact use the same grading system is an area for future research.

Third, ERFs are best addressed early, and not during application deliberations when “damage” has already been done. This may mean encouraging preventive measures at the design stage of clinical trials, widening the capacity of research ethics committees to monitor approved clinical trials, reviewing sponsor responsibility in actively pursuing ethically compliant trials, and/or more active collaboration between RECs and drug regulators in terms of approving and monitoring clinical trials, among others.

Fourth, inspection reports provide a lot of insight on ethical and scientific matters such as the ethical acceptability of the elements of a pharmaceutical clinical trial which eventually becomes a basis for an application, integrity of the clinical trial data based on which pharmaceutical products are provided marketing authorization, among others. This should be sufficient reason for drug regulatory agencies to make them more accessible, if not public. This is a concern that was earlier made by Dal-Re, Kesselheim, and Bourgeois [12] in an opinion piece calling for the publication of inspection reports by drug regulatory bodies. Dal-re and colleagues correctly point out that doing so is part of these regulatory bodies’ public health mandate. It also allows for (a) individual assessment of “trial quality in publication decisions”; (b) provides more inputs for systematic reviews; and (c) provide means for clinical trial sponsors to correct mistakes and ensure participant safety [12].

Fifth, we saw above the EMA position that GCP issues, even those that do not affect the benefit-risk balance so long as these issues raise “serious questions about the rights, safety, and well-being of trial subjects” should have an “impact on the final conclusions about approvability of an application” [4]. In our study, we found that this is not (yet) the case. Unfortunately, we found no EMA document that elaborates on how ethical issues should affect application evaluation processes and no other publication to our knowledge engaged these issues, except ours. In an earlier publication [13], we proposed a 4-step procedure in evaluating ERFs, with sanctions depending on the evaluation of the gravity and magnitude of the ERF. However, it still remains to be seen how ERFs that do not affect the risk–benefit balance of an application such as the ones we dealt with in this manuscript should be evaluated by assessors and how such an assessment should impact the assessment process. This is work for future research.

## Conclusion

None of the ethically relevant findings, all of which were graded as major or critical in integrated inspection reports, were explicitly carried over to the joint assessment reports. This means that *from the vantage of these joint assessment reports*, none of the ethically relevant findings seemed to have reached or impacted the application deliberation processes. This calls for more transparency in EMA application deliberations, specifically on how ERFs are considered in the decision-making processes.

## Data Availability

The inspection reports, integrated inspection reports, Day 150, and Day 180 data that support the findings of this study are available from the database of the Dutch Medicines Evaluation Board (MEB). Because of the sensitivity of the sources, the data may only be accessed with the permission of the MEB or similar European regulatory body.

## Abbreviations

CHMP: Committee for Medicinal Products for Human Use
CAPA: Corrective and preventive action
ERFs: Ethically relevant findings
EMA: European Medicines Agency
GCP: Good Clinical Practice
IC: Informed consent
REC: Research ethics committees
